# Characterization and Genome Analysis of *Arthrobacter bangladeshi* sp. nov., Applied for the Green Synthesis of Silver Nanoparticles and Their Antibacterial Efficacy against Drug-Resistant Human Pathogens

**DOI:** 10.3390/pharmaceutics13101691

**Published:** 2021-10-15

**Authors:** Md. Amdadul Huq, Shahina Akter

**Affiliations:** 1 Department of Food and Nutrition, College of Biotechnology and Natural Resource, Chung-Ang University, Anseong 17546, Korea; 2 Department of Food Science and Biotechnology, Gachon University, Seongnam 461-701, Korea

**Keywords:** *Arthrobacter bangladeshi* MAHUQ-56^T^, AgNPs, green synthesis, *S. typhimurium*, *Y. enterocolitica*

## Abstract

The present study describes the isolation and characterization of novel bacterial species *Arthrobacter bangladeshi* sp. nov., applied for the green synthesis of AgNPs, and investigates its antibacterial efficacy against drug-resistant pathogenic *Salmonella* Typhimurium and *Yersinia enterocolitica*. Novel strain MAHUQ-56^T^ is Gram-positive, aerobic, non-motile, and rod-shaped. Colonies were spherical and milky white. The strain showed positive activity for catalase and nitrate reductase, and the hydrolysis of starch, L-tyrosine, casein, and Tween 20. On the basis of the 16S rRNA gene sequence, strain MAHUQ-56^T^ belongs to the *Arthrobacter* genus and is most closely related to *Arthrobacter pokkalii* P3B162^T^ (98.6%). *Arthrobacter bangladeshi* MAHUQ-56^T^ has a genome 4,566,112 bp long (26 contigs) with 4125 protein-coding genes, 51 tRNA and 6 rRNA genes. The culture supernatant of *Arthrobacter bangladeshi* MAHUQ-56^T^ was used for the easy and green synthesis of AgNPs. Synthesized AgNPs were characterized by UV–vis spectroscopy, FE-TEM, XRD, DLS, and FT-IR. Synthesized AgNPs were spherical and 12–50 nm in size. FT-IR analysis revealed various biomolecules that may be involved in the synthesis process. Synthesized AgNPs showed strong antibacterial activity against multidrug-resistant pathogenic *S. typhimurium* and *Y. enterocolitica*. MIC values of the synthesized AgNPs against *S. typhimurium* and *Y. enterocolitica* were 6.2 and 3.1 ug/mL, respectively. The MBC of synthesized AgNPs for both pathogens was 12.5 ug/mL. FE-SEM analysis revealed the morphological and structural alterations, and damage of pathogens treated by AgNPs. These changes might disturb normal cellular functions, which ultimately leads to the death of cells.

## 1. Introduction

The *Arthrobacter* genus within the *Micrococcaceae* family was first proposed by Conn and Dimmick [1]. The *Arthrobacter* genus comprises 54 validly published species at the time of writing (https://lpsn.dsmz.de/genus/Arthrobacter (accessed on 28 August 2021)). Members of this genus are Gram-positive, aerobic (requiring oxygen for growth), nonmotile, rod-coccus-shaped, with negative acidification of glucose, catalase-positive, and contain C_15:0 anteiso_, C_15:0 iso_, C_16:0 iso_ and C_17:0 anteiso_ as the major fatty acids, MK-9 (H2) or MK-8/MK-9 as the predominant respiratory quinone, and high DNA G + C content [2,3]. The *Arthrobacter* genus is one of the most divergent heterotrophic bacterial groups because of metabolic versatility. Members of the *Arthrobacter* genus were isolated from various environments, such as sewage, soil, water, ice, rhizosphere, marsh, sediment, and pests [4,5,6,7,8]. Many species of this genus were utilized for beneficial purposes [4,5,7]. Here, a novel species of *Arthrobacter* was isolated from the soil sample of a rice field and applied for the green synthesis of silver nanoparticles (AgNPs). On the basis of the phylogenetic analysis of 16S rRNA gene sequence, genome sequence analysis, digital DNA–DNA hybridization analysis, and physiological and chemotaxonomic characteristics, we demonstrate that strain MAHUQ-56^T^ represents a novel species of the *Arthrobacter* genus, for which the name *Arthrobacter bangladeshi* sp. nov. (type strain MAHUQ-56^T^) is proposed.

Nanotechnology deals with the design, synthesis, and application of nanosized particles in the range of 1–100 nm. There are different kinds of metallic nanoparticles, such as gold, silver, iron, and zinc. Among them, AgNPs are widely used nanoparticles due to their various applications in biological fields such as antimicrobial, anticancer, drug delivery, catalysis, and biomolecular detection [9,10,11]. Due to the various applications of metal nanoparticles in different scientific fields, researchers have devoted extensive efforts to develop easy and ecofriendly techniques for the rapid and mass production of nanoparticles. Chemical, physical, and biological methods are available for the synthesis of nanoparticles [12]. Chemical and physical methods are commonly used for the synthesis of well-characterized nanoparticles. However, these two method types need enormous energy amounts and hazardous chemicals, and produce toxic byproducts that make them unsuitable approaches [13,14]. Therefore, the development of a green approach for the synthesis of nanoparticles is an emerging demand to avoid the drawbacks of conventional physical and chemical methods. Biological methods are an important route for the synthesis of nanoparticles because of their nontoxic, cost-effective, and ecofriendly properties [15,16]. For the ecofriendly synthesis of nanoparticles, various biological resources could be used, such as plants or plant products, and microorganisms such as bacteria, fungi, and algae [13,16,17,18,19]. Among different natural resources, bacteria are mostly chosen for the biosynthesis of nanoparticles because of their easy handling and growth, and large-scale production [20,21].

The emergence of multidrug-resistant bacteria is a major concern for public health. Antibiotic-resistant microorganisms cause life-threatening diseases in humans. *Salmonella* Typhimurium is a rod-shaped, aerobic, flagellated, Gram-negative bacterium, and a primary enteric pathogen for both humans and animals. Infection begins with the ingestion of contaminated food or water. After consuming the contaminated food or water, the pathogen reaches the intestinal epithelium and triggers gastrointestinal disease [22]. *Yersinia enterocolitica* is a Gram-negative, rod-shaped bacterium within the *Yersiniaceae* family. Yersiniosis disease is developed by *Y. enterocolitica* infection, which is an animal-borne disease occurring in humans and different animals such as cattle, pigs, deer, and birds [23]. Both *S. typhimurium* and *Y. enterocolitica* showed resistance against various drugs [24,25]. The development of a novel antibacterial agent is the key solution for this issue. Therefore, green synthesized AgNPs are a promising agent to control these multidrug-resistant bacteria. The present study isolates and characterizes novel bacterial species *Arthrobacter bangladeshi* sp. nov., used for the easy and ecofriendly extracellular synthesis of AgNPs, and investigates its antibacterial efficacy against drug-resistant pathogenic *S. typhimurium* and *Y. enterocolitica*.

## 2. Materials and Methods

### 2.1. Materials

Silver nitrate (AgNO_3_) and bacterial growth media were obtained from Sigma-Aldrich Chemicals (St. Louis, MO, USA). Pathogenic strains *Salmonella* Typhimurium (ATCC 14028) and *Yersinia enterocolitica* (ATCC 9610) were collected from American Type Culture Collection (ATCC).

### 2.2. Isolation of AgNP-Producing Bacteria

Strains were isolated from the soil sample of a rice field located in Dighalgram, Magura, Bangladesh. Samples were collected in 15 mL conical tubes and suspended in sterile NaCl solution (0.85%, *w*/*v*). Strains were isolated through a serial dilution technique using an R2A agar medium after incubation for 3 days at 30 °C according to a previous study [18]. To check the AgNPs’ synthesis ability, all isolated strains were separately cultured in 5 mL R2A broth media for 72 h at 30 °C. Then, the culture supernatant was collected and incubated with 1 mM AgNO_3_ solution (final concentration) in a shaking incubator for 72 h at 30 °C. On the basis of AgNO_3_ reduction efficacy to AgNPs, strain MAHUQ-56^T^ was selected as the perfect candidate. Strain MAHUQ-56^T^ was stored at −80 °C in R2A broth containing 30% (*v*/*v*) glycerol. Strain MAHUQ-56^T^ was deposited into the Korean Agriculture Culture Collection (KACC) and China General Microbiological Culture Collection Center (CGMCC).

### 2.3. Phenotypic, Physiological, and Biochemical Characteristics

The growth of strain MAHUQ-56^T^ was investigated at 30 °C for 5 days on several agar media, namely, R2A agar, nutrient agar (NA), MacConkey agar, Luria–Bertani (LB) agar, and trypticase soy agar (TSA). To determine the optimal growth temperature, strain MAHUQ-56^T^ was cultivated at different temperatures (5–45 °C with 5 °C intervals) on R2A agar. To investigate the optimal growth pH, strain MAHUQ-56^T^ was cultivated at different pH values (3.0–11.0 at intervals of 0.5 pH unit) in an R2A broth medium. NaCl tolerance was examined in R2A broth medium (0–5% (*w*/*v*)). Gram staining (bioMérieux) was examined according to the manufacturer’s instructions. Cell morphology was assessed by transmission electron microscopy after culturing the strain for 2 days at 30 °C on an R2A agar medium. The cell motility and anaerobic growth ability of strain MAHUQ-56^T^ were established according to a previous description [18]. The production of flexirubin-type pigments was checked according to Sheu et al. [26]. The activities of DNase, oxidase, urease, and catalase, and the hydrolysis of starch, gelatin, casein, and Tweens 20 and 80 were found according to a previous description [18]. Additional biochemical and physiological characterization of strain MAHUQ-56^T^ and close relatives was investigated using the API ZYM and API 20NE kits (bioMérieux). Close type strains *Arthrobacter pokkalii* KCTC 29498^T^, *Pseudarthrobacter defluvii* KCTC 19209^T^, *Pseudarthrobacter niigatensis* CCTCC AB 206012^T^, *Pseudarthrobacter phenanthrenivorans* DSM 18606^T^, and *Pseudarthrobacter enclensis* DSM 25279^T^ were used as reference strains, and grown under the same experimental conditions to compare phenotypic properties and fatty acid compositions.

### 2.4. 16S rRNA Gene Sequencing and Phylogenetic Analysis

Phylogenetic analysis of isolated strain MAHUQ-56^T^ was conducted using the 16S rRNA gene sequence. Having extracted the genomic DNA of strain MAHUQ-56^T^, the 16S rRNA gene was amplified by PCR using bacterial universal primers 27F and 1492R [27]. The PCR product was purified and sequenced by Biofact Co. Ltd. (Daejeon, South Korea). The 16S rRNA sequence similarity was checked and compared with validly published type strains from the EzTaxon database (http://www.ezbiocloud.net/eztaxon (accessed on 28 August 2021)) [28]. Phylogenic trees were constructed using neighbor-joining and maximum-likelihood algorithms [26] in MEGA software, Version 6 [29] with bootstrap values of 1000 replications [30].

### 2.5. Genomic Sequence Analysis

The draft genomic sequence of strain MAHUQ-56^T^ was analyzed by Illumina HiSeq X Ten (Illumina, Inc., San Diego, CA, USA), and assembly was carried out using a de novo assembler (SOAPdenovo v. 3.10.1). The NCBI prokaryotic genome annotation pipeline (PGAP) was used for genome annotation. DNA G + C content of strain MAHUQ-56^T^ was directly calculated from its genomic sequence. Average nucleotide identity (ANI) values were analyzed to investigate the level of pairwise relatedness between MAHUQ-56^T^ and close type strains using the EzTaxon-e server (https:// www.ezbio cloud.net/tools/ani (accessed on 28 August 2021)) [28]. Digital DNA–DNA hybridization (dDDH) values were determined using the genome-to-genome distance calculator (http://ggdc.dsmz.de/ggdc.php (accessed on 28 August 2021)) [31]. For functional analysis, the genome of strain MAHUQ-56^T^ was annotated using the Rapid Annotation Subsystems Technology (RAST) server (https://rast.nmpdr.org (accessed on 28 August 2021)) [32].

### 2.6. Cellular Fatty Acid and Respiratory Quinones Analysis

To identify fatty acid compositions, strain MAHUQ-56^T^ and reference strains were cultured on an R2A agar medium at 30 °C for 48 h. Extraction, purification, and analysis of cellular fatty acids were performed according to a previous description [18,33]. The respiratory quinones of strain MAHUQ-56^T^ were extracted, purified, and analyzed by HPLC using the method of Collins [34].

### 2.7. Biosynthesis of AgNPs Using Arthrobacter bangladeshi sp. nov.

The green synthesis of AgNPs was performed using the culture supernatant of strain MAHUQ-56^T^. Briefly, strain MAHUQ-56^T^ was cultured in 100 mL R2A broth medium for 3 days at 30 °C with 180 rpm. Then, the bacterial culture was spun down at 9000 rpm for 10 min to obtain a cell-free supernatant. We added 1 mM of AgNO_3_ solution (final concentration) to the 100 mL cell-free culture supernatant and incubated it again in dark shaking condition (180 rpm, 35 °C) for 48–72 h. The reaction mixture was observed continuously for the synthesis of nanoparticles by visual inspection and UV–vis spectral analysis. Synthesized AgNPs were collected by high-speed centrifugation (14,000 rpm for 20 min). Collected AgNPs were washed several times with distilled water and obtained in precipitate form. Precipitated AgNPs were air-dried, and used for characterization and antimicrobial application.

### 2.8. Characterization of Synthesized AgNPs

The UV–vis spectrum of synthesized AgNPs was analyzed by UV–vis spectrophotometer (Optizen POP, Mecasys, Daejeon, South Korea) in the range of 300–800 nm. To investigate the morphology, composition, and metallic nature of synthesized AgNPs, field emission-transmission electron microscopy (FE-TEM) analysis was conducted, operated with voltage of 200 kV (JEM 2100F, JEOL, Tokyo, Japan). An AgNP sample for TEM was prepared by dissolving in distilled water, and spotting onto carbon-coated TEM grid, followed by air-drying at room temperature. The air-dried AgNP sample was used for X-ray diffraction (XRD) analysis using an X-ray diffractometer (D8 Advance, Bruker, Germany) over a 2θ value in the range of 30°–90°. The hydrodynamic diameters and polydispersity index of green synthesized AgNPs were investigated by dynamic light scattering (DLS, Otsuka Electronics, Osaka, Japan) (Malvern Zetasizer Nano ZS90) according to a previous description [35]. Fourier transform-infrared (FTIR, PerkinElmer Inc., Waltham, MA, USA) analysis was performed by scanning the air-dried purified AgNPs using FTIR spectroscopy over the range of 4000–500 cm^−1^.

### 2.9. Antimicrobial Activity of Synthesized AgNPs

The antibacterial properties of green synthesized AgNPs against pathogenic *S. typhimurium* and *Y. enterocolitica* were investigated by the disc diffusion method. In brief, the tested pathogens were grown overnight in Mueller–Hinton (MH) broth. Then, 100 μL culture suspensions of both *S. typhimurium* and *Y. enterocolitica* were spread onto MH agar plates, and 8 mm sterile paper discs were placed on the surface of MH agar plates. Then, 30 and 60 μL (1000 ppm) of green synthesized AgNPs solutions (AgNPs were dissolved in water) were placed onto the paper discs. Plates were incubated at 37 °C for 24 h to observe the inhibition zones. The inhibition zone was calculated in millimeters (mm) after 24 h of incubation. This test was performed three times.

### 2.10. MIC and MBC Investigation

The bacteriostatic activity of green synthesized AgNPs was determined by measuring minimum inhibitory concentration (MIC) using microdilution assay as follows. Pathogenic strains *S. typhimurium* and *Y. enterocolitica* were grown in MH broth medium at 37 °C overnight; then, the culture was diluted to approximately 1 × 10^6^ CFUs/mL. Then, 100 uL of diluted bacterial culture was added into 96-well ELISA plate, followed by an equal volume of AgNPs solutions (AgNPs were dissolved in MH broth medium) with various concentrations (3.1, 6.2, 12.5, 25, 50, 100, and 200 μg/mL). As a control, only MH broth was used instead of an AgNP solution. Samples were incubated at 37 °C for 24 h. Every 3 h of the interval, bacterial growth was calculated using an ELISA plate reader (LabTech 4000, BMG LABTECH, Ortenberg, Germany) by recording OD_600_. Minimum bactericidal concentration (MBC) was defined as the lowest concentration of synthesized AgNPs that was required to kill the tested pathogenic strains. For measuring MBC, 10 μL of the above mixtures was streaked on MH agar plates and incubated at 37 °C for 24 h. Lastly, MBC was evaluated by determining the lowest concentration that killed the bacteria [36].

### 2.11. Investigation of Morphological Changes by FE-SEM

Bacterial strains of *S. typhimurium* and *Y. enterocolitica* (approximately 1 × 10^7^ CFU/mL) were each incubated with and without biosynthesized AgNPs (at MBC concentration) at 37 °C overnight. Upon the end of the incubation period, samples were processed for FE SEM analysis to investigate structural changes according to a previous report [36]. AgNP-treated cells were collected by centrifugation at 8000 rpm for 5 min. Then, cells were washed by PBS (pH 7.0) and fixed by 2.5% glutaraldehyde for 4 h at room temperature. Cells were again washed by PBS and serially dehydrated by various concentrations of ethanol (from 30% to 100%) in 10 min intervals at room temperature. Subsequently, dehydrated cells were dried using a desiccator, and samples were lastly coated with gold for the investigation of structural changes by FE-SEM (S-4700, Hitachi, Tokyo, Japan).

## 3. Results and Discussion

### 3.1. Phenotypic, Physiological, and Biochemical Characteristics

**Figure 1:**
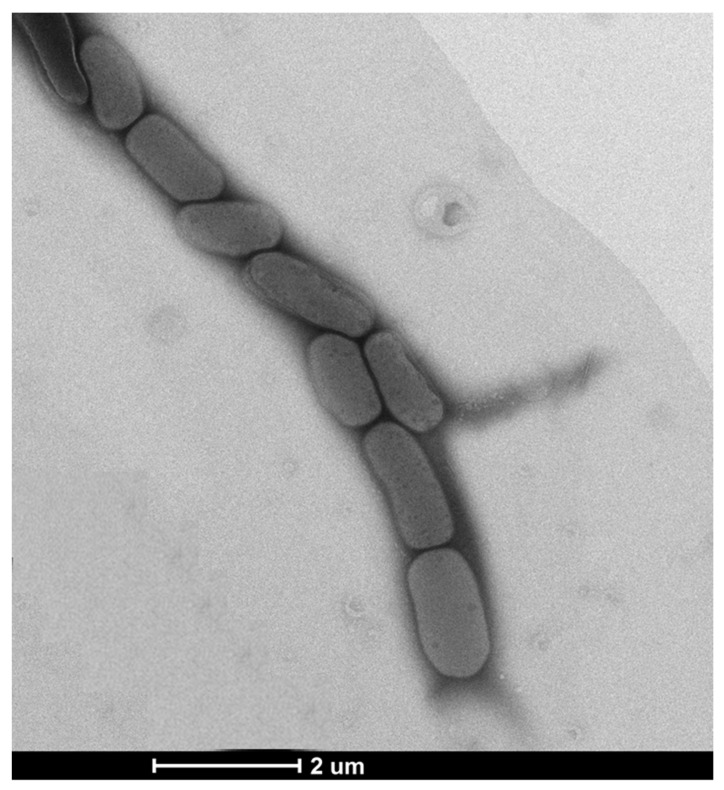
Transmission electron micrograph of cells of *Arthrobacter bangladeshi* MAHUQ-56^T^ after negative staining with uranyl acetate; Bar, 2.0 μm.

Cells of strain MAHUQ-56^T^ were Gram-positive, aerobic, non-motile, and rod-shaped, 0.6–1.0 μm wide and 1.3–2.5 μm long (Figure 1). Strain MAHUQ-56^T^ grew on R2A agar, NA, TSA, and LB agar media, but not on MacConkey agar. Colonies were spherical, milky white, and 0.4–0.9 mm in diameter when grown on R2A agar medium for 2 days. The strain showed positive activity for catalase, but negative for oxidase- and flexirubin-type pigments. Cells could hydrolyze starch, L-tyrosine, casein and Tween 20, but were negative for DNA, gelatin, esculin, urea, and Tween 80. Strain MAHUQ-56^T^ was positive for the assimilation of glucose, mannose, arabinose, maltose, gluconate, N-acetyl-glucosamine, malate, citrate, mannitol, adipate, and phenyl-acetate (API 20NE). Based on the API ZYM kit, strain MAHUQ-56^T^ was positive for valine arylamidase, esterase, acid phosphatase, esterase lipase (C8), trypsin, alkaline phosphatase, leucine arylamidase, cystine arylamidase, *β*-glucosidase, naphthol-AS-BI-phosphohydrolase, *β*-galactosidase, *α*-glucosidase, *α*-mannosidase, *α*-galactosidase, *α*-chymotrypsin, *α*-fucosidase, *β*-glucuronidase and lipase (C14). Strain MAHUQ-56^T^ showed many different phenotypic characteristics from its close relatives, including different growth conditions, and enzymatic and assimilation activities (Table 1). Strain MAHUQ-56 was deposited into KACC (KACC 22003^T^) and CGMCC (CGMCC 1.18517^T^).

### 3.2. 16S rRNA Gene Sequence and Phylogenetic Analysis

**Figure 2:**
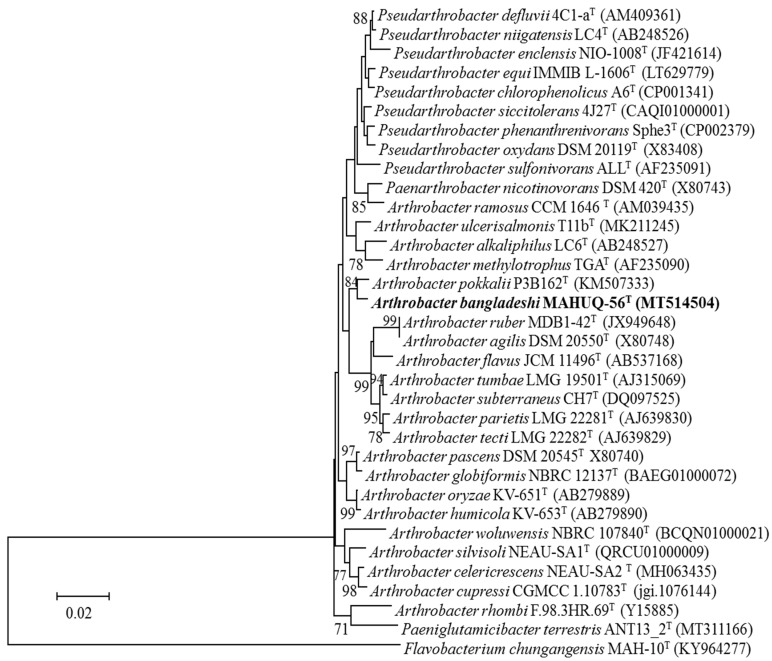
Neighbor-joining (NJ) tree based on 16S rRNA gene sequence analysis showing phylogenetic relationships of strain MAHUQ-56 and the related type strains. Bootstrap values more than 70% based on 1000 replications shown at branching points. Scale bar, 0.02 substitutions per nucleotide position.

The 16S rRNA gene sequence (1451 bp, GenBank accession number, MT514504) similarities showed that strain MAHUQ-56^T^ was most closely related to *Arthrobacter pokkalii* P3B162^T^ (98.6%), *Pseudarthrobacter defluvii* 4C1-a^T^ (98.5%), *Pseudarthrobacter niigatensis* LC4^T^ (98.2%), *Pseudarthrobacter phenanthrenivorans* Sphe3^T^ (98.2%), and *Pseudarthrobacter enclensis* NIO-1008^T^ (98.0%). Phylogenetic analysis based on 16S rRNA gene sequences using neighbor-joining (NJ) (Figure 2) and maximum-likelihood (ML) (Supplementary Figure S1) phylogenetic trees revealed that strain MAHUQ-56^T^ clustered within the *Arthrobacter* genus and formed a monophyletic clade to *Arthrobacter pokkalii*. From 16s rRNA gene sequence and phylogenetic analysis, it is clear that strain MAHUQ-56^T^ is a new member of the *Arthrobacter* genus.

### 3.3. Draft Genome and DNA G + C Content Analysis

**Figure 3:**
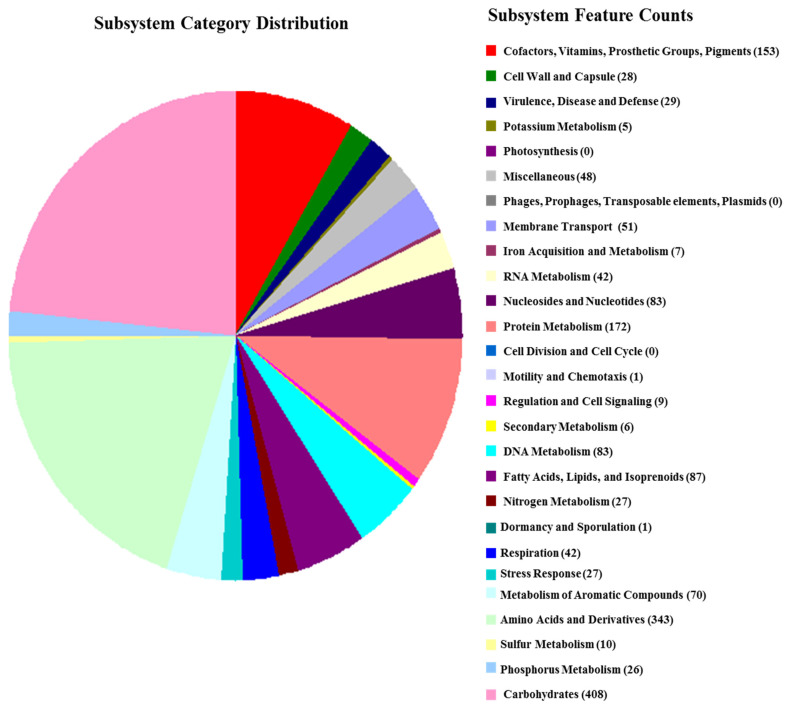
COG functional genes of *Arthrobacter bangladeshi* MAHUQ-56^T^.

The draft genome of strain MAHUQ-56^T^ contains 26 contigs with an N50 size of 360,503 bp. Total genome size was 4,566,112 bp, with an average G + C content of 66.0 mol%. Gene annotation by PGAP revealed 4125 protein-encoding genes, 51 tRNA genes, and 6 rRNA genes (NCBI accession number, JAIFZQ000000000). The common features of the genome sequence of strain MAHUQ-56^T^ are shown in Table 2. Characterization of novel species is increasingly dependent on genomic sequence [37]. Functional annotation by the RAST server showed that 172 genes were involved with the metabolism of proteins, 83 genes were associated with the metabolism of nucleosides and nucleotides, 343 genes were linked with amino acids and derivatives, and 408 genes were involved with carbohydrate metabolism (Figure 3). The closest type strain, *Arthrobacter pokkalii* GZK-1, contains a 4,415,912 bp long genome with 65.9 mol% GC, 4091 protein-encoding genes, 15 rRNA genes, and 51 tRNA genes (https://www.ezbiocloud.net/genome/explore?puid=230640 (accessed on 28 August 2021)). Genomic ANI values between strain MAHUQ-56^T^ and close type strains *A. pokkalii* GZK-1^T^, *P. phenanthrenivorans* Sphe3^T^ and *P. enclensis* NIO-1008^T^ were 84.0%, 81.5%, and 82.0%, respectively (Supplementary Table S1), which were well below (≥95–96%) suggesting a novel species. The dDDH values based on the draft genomes between strain MAHUQ-56^T^ and close relatives *A. pokkalii* GZK-1^T^, *P. phenanthrenivorans* Sphe3^T^ and *P. enclensis* NIO-1008^T^ were 27.5%, 24.5%, and 24.8%, respectively (Supplementary Table S1), which were also far below the threshold value (70%) for species delineation.

### 3.4. Cellular Fatty Acid and Respiratory Quinones Analysis

The predominant fatty acids of strain MAHUQ-56^T^ were C_15:0 anteiso_ (47.8%), C_15:0 iso_ (11.9%), C_16:0 iso_ (15.8%), and C_17:0 anteiso_ (11.7%). Overall fatty acid composition was similar to those of close type strains. However, there were some clear quantitative and qualitative differences in the fatty acid profiles (Table 3). The major respiratory quinone of strain MAHUQ-56^T^ was MK-9(H2). Closest type strain *Arthrobacter pokkalii* P3B162^T^ also showed the same major respiratory quinone [4].

### 3.5. Taxonomic Conclusions

Phenotypic, chemotaxonomic, and biochemical characteristics, and phylogenetic inference and genomic features support that strain MAHUQ-56^T^ represents a novel species of the *Arthrobacter* genus, for which the name *Arthrobacter bangladeshi* sp. nov. is proposed.

### 3.6. Description of Arthrobacter bangladeshi sp. nov.

*Arthrobacter bangladeshi* (bangla.desh′i. N.L. gen. n. *bangladeshi* pertaining to Bangladesh, where the rice field is located and the type strain was isolated).

Cells are Gram-stain-positive, aerobic, non-motile, and rod-shaped, 0.6–1.0 μm wide and 1.3–2.5 μm long. Strain MAHUQ-56^T^ grew on R2A agar, NA, TSA, and LB agar media, but not on MacConkey agar. Colonies were spherical, milky white, and 0.4–0.9 mm in diameter when grown on R2A agar medium for 2 days. Growth occurs at 10–40 °C (optimum, 30  °C), at pH 5.0–10.0 (optimum, pH 7.0) and with 0–2% NaCl (optimum, 0%). Positive for catalase activity and the hydrolysis of starch, L-tyrosine, casein and Tween 20. Negative for oxidase, flexirubin-type pigments production, glucose fermentation, and the hydrolysis of DNA, gelatin, esculin, urea, and Tween 80. Nitrate is reduced to nitrite. Strain MAHUQ-56^T^ was positive for the assimilation of glucose, mannose, arabinose, maltose, gluconate, N-acetyl-glucosamine, malate, citrate, mannitol, adipate and phenyl-acetate, but negative for the assimilation of caprate (API 20NE). In API ZYM tests, valine arylamidase, C4 esterase, acid phosphatase, esterase lipase (C8), trypsin, alkaline phosphatase, leucine arylamidase, cystine arylamidase, *β*-glucosidase, naphthol-AS-BI-phosphohydrolase, *β*-galactosidase, *α*-glucosidase, *α*-mannosidase, *α*-galactosidase, *α*-chymotrypsin, *α*-fucosidase, *β*-glucuronidase and lipase (C14) activities were present, but N-acetyl-*β*-glucosaminidase activities were absent. Major fatty acids were C_15:0 anteiso_, C_15:0 iso_, C_16:0 iso_ and C_17:0 anteiso_. The predominant respiratory quinone was MK-9(H2). The DNA G + C content of the type strain MAHUQ-56^T^ was 66.0 mol%.

Type strain was MAHUQ-56^T^ (=KACC 22003^T^ = CGMCC 1.18517^T^), isolated from soil sample of a rice field, Magura, Bangladesh.

### 3.7. Green Synthesis of AgNPs Using Arthrobacter bangladeshi sp. nov.

**Figure 4:**
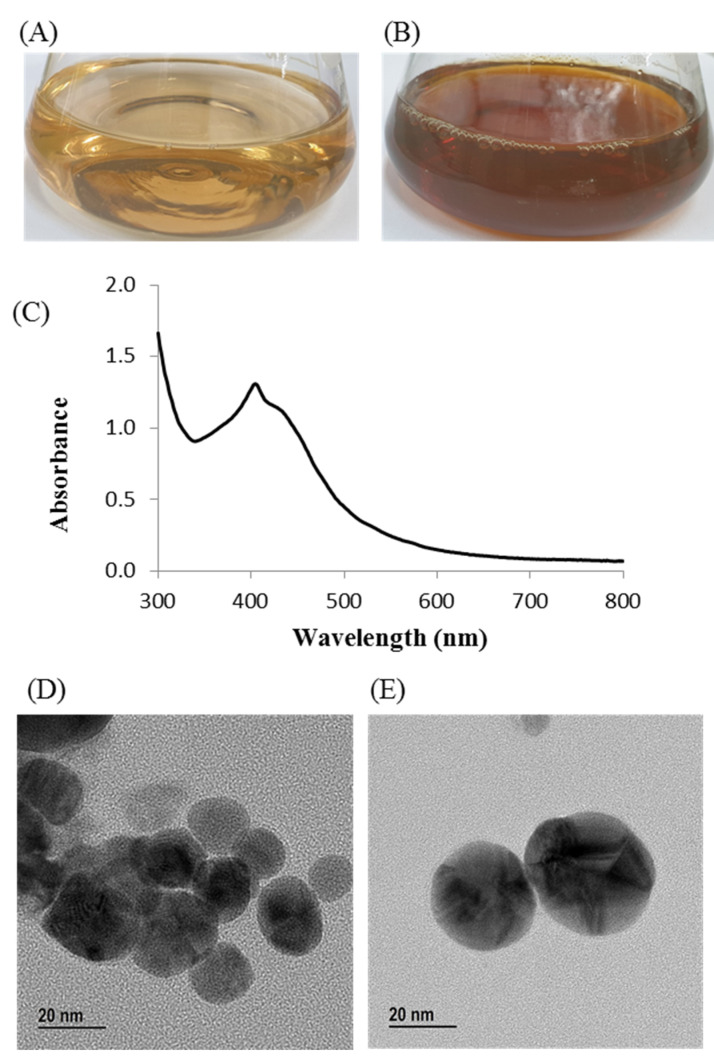
(**A**) R2A broth with AgNO_3_ as control, (**B**) biosynthesized AgNPs, (**C**) UV–vis spectra, and (**D**,**E**) FE-TEM images of synthesized AgNPs.

The culture supernatant of *Arthrobacter bangladeshi* MAHUQ-56^T^ was supplemented with 1 mM AgNO_3_ for the green synthesis of AgNPs. The synthesis of AgNPs by strain MAHUQ-56^T^ was initially confirmed by visual observation. The color of the reaction mixture changed into deep brown from pale yellow within 72 h of incubation (Figure 4B). The change in color is attributed to the surface plasmon resonance of AgNPs, and indicated the formation of AgNPs [38,39]. There was no color change in the control (Figure 4A). Intracellular and extracellular methods are available for the bacteria-mediated synthesis of AgNPs. The extracellular method is easier and more rapid compared to the intracellular method, which needs complex purification steps [40]. In the current study, a facile and convenient extracellular process was used for the biosynthesis of AgNPs using *Arthrobacter bangladeshi* MAHUQ-56^T^.

### 3.8. Characterization of Green Synthesized AgNPs

**Figure 5:**
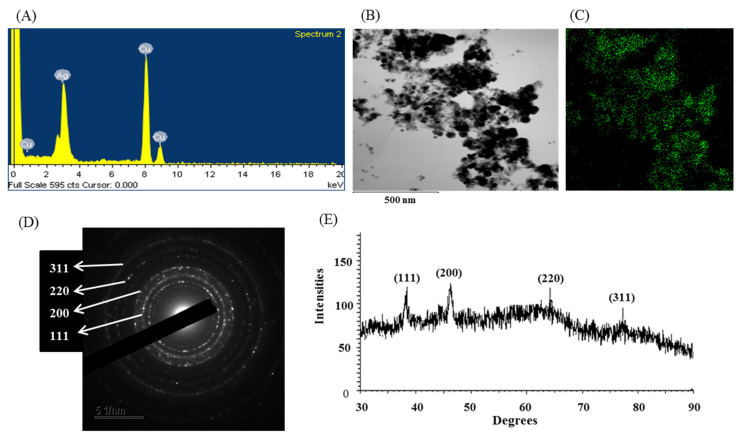
(**A**) EDX spectrum of synthesized AgNPs, (**B**) TEM image used for mapping, (**C**) distribution of silver in elemental mapping, (**D**) SAED pattern, and (**E**) X-ray diffraction pattern of biosynthesized AgNPs.

The biosynthesis of AgNPs was further confirmed by UV–visible spectral analysis in the range of 300–800 nm. A strong peak appeared at 405 nm (Figure 4C), which ensured the synthesis of AgNPs [41]. Our results agree with those of Du et al., who reported that AgNPs synthesized by *Novosphingobium* sp. THG-C3 showed an SPR band at 406 nm [35]. The standard SPR peak of biosynthesized Ag-NPs generally shows within 400 to 450 nm [42]. The morphology of synthesized AgNPs was investigated by FE-TEM, which revealed a spherical shape. The size of *Arthrobacter bangladeshi* MAHUQ-56^T^-mediated synthesized AgNPs was determined to the range of 12 to 50 nm (Figure 4D,E). Singh et al. reported a similar particle size (10–40 nm) of AgNPs that was synthesized by *Cedecea* sp. [43]. The elemental composition and purity of synthesized AgNPs was examined by EDX, as shown in Figure 5. The EDX spectrum revealed a strong peak for silver at 3 keV, which ensures the synthesis of metallic AgNPs [44]. Some extra peaks were also found in the EDX spectrum due to the use of copper grids (Figure 5A). Elemental mapping results showed the highest distribution of silver elements in the sample (Figure 5B,C, Table 4). To investigate the phase of AgNPs, selected area electron diffraction (SAED) analysis was performed (Figure 5D). SAED analysis showed a sharp rings corresponding to the following reflections at 111, 200, 220, and 311 of lattice planes (Figure 5D), which indicated the crystalline nature of biosynthesized AgNPs. X-ray diffraction (XRD) analysis was conducted to determine the crystalline property of synthesized AgNPs. The XRD pattern showed four distinct peaks at 2θ values of 38.21°, 46.42°, 64.69°, and 77.41° corresponding to the intensities of 111, 200, 220, and 311 for the reflections of metallic silver (Figure 5E). Some recently reported studies showed similar XRD patterns of AgNPs synthesized by microorganisms [35,45].

**Figure 6:**
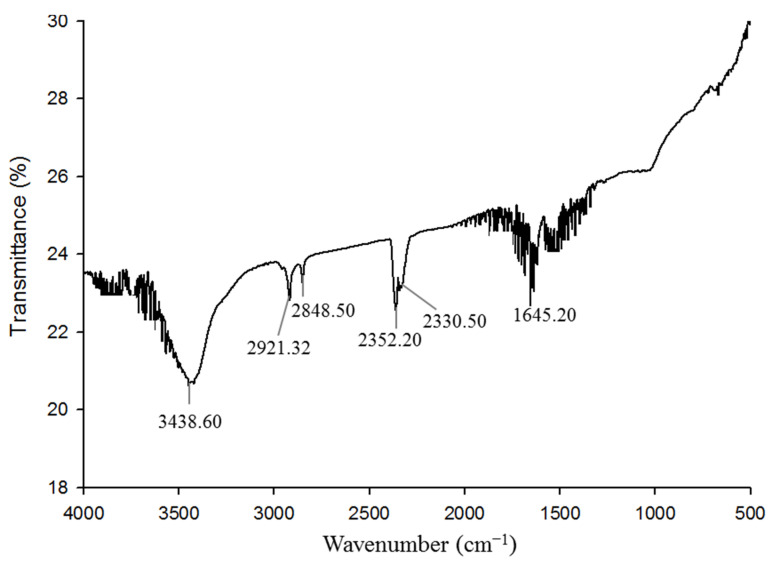
FT-IR spectra of *Arthrobacter bangladeshi* MAHUQ-56^T^-mediated synthesized AgNPs.

The size and polydispersity value of the synthesized AgNPs was investigated by DLS. The average hydrodynamic diameter was 122.5 nm, with a polydispersity index of 0.286 (Supplementary Figure S2). The possible role of biomolecules presence in bacterial culture supernatant for the synthesis and stabilization of AgNPs was identified by Fourier transform infrared (FT-IR) analysis. The FTIR spectrum revealed major intense absorbance peaks at 3438.60, 2921.32, 2848.50, 2352.20, 2330.50, and 1645.20 cm^−1^ (Figure 6). The vibration peak at 3438.60 cm^−1^ may have been due to the presence of an O–H (alcohol) and/or N–H (amine) group with synthesized AgNPs. The bands at 2921.32 and 2848.50 cm^−1^ were due to the stretching vibration of C-H (alkane) group. Absorbance peaks at 2352.20 and 2330.50 cm^−1^ could have been the presence of O=C=O (carbonyl bond group). The band located at 1645.20 cm^−1^ may have been due to the presence of a C=O (ester) or –C=C bond. FT-IR data identified the presence of biomolecules, which may have been responsible for the synthesis and stabilization of AgNPs. Some previous studies showed a similar FT-IR spectrum of AgNPs synthesized using microorganisms [43,46]. Supplementary Table S2 shows the physicochemical properties of AgNPs synthesized by *Arthrobacter bangladeshi* and some other bacterial strains.

### 3.9. Antimicrobial Activity of Synthesized AgNPs

**Figure 7:**
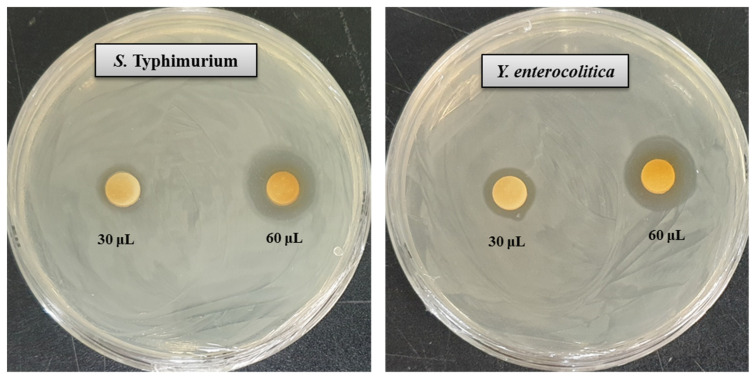
Zones of inhibition of 30 and 60 μL of biosynthesized AgNPs at 1000 ppm concentrations in water against *S. typhimurium* and *Y. enterocolitica*.

In the present study, the antibacterial activity of *Arthrobacter bangladeshi* MAHUQ-56^T^-mediated synthesized AgNPs was investigated against multidrug-resistant pathogenic *S. typhimurium* and *Y. enterocolitica.* Synthesized AgNPs exhibited strong antibacterial activity against both tested pathogens *S. typhimurium* and *Y. enterocolitica*. The potential antibacterial activity of synthesized AgNPs against pathogenic microorganisms was confirmed by the formation of an inhibition zone (Figure 7). Figure 7 reveals a clear zone of inhibition (ZOI) around the paper discs treated by synthesized AgNPs. The diameters of the ZOI of biosynthesized AgNPs *against* *S. typhimurium* and *Y. enterocolitica* were 18.3 ± 0.6 and 20.4 ± 0.8 mm, respectively, and are shown in Table 5. The biosynthesized AgNPs showed the highest activity against multidrug-resistant *Y. enterocolitica,* followed by *S. typhimurium*. These results suggest that *Arthrobacter bangladeshi* MAHUQ-56^T^-mediated synthesized AgNPs could be useful as an antibacterial agent to control multidrug-resistant *S. typhimurium* and *Y. enterocolitica*. These findings were consistent with those of previous studies [35,45].

### 3.10. MIC and MBC Investigation

**Figure 8:**
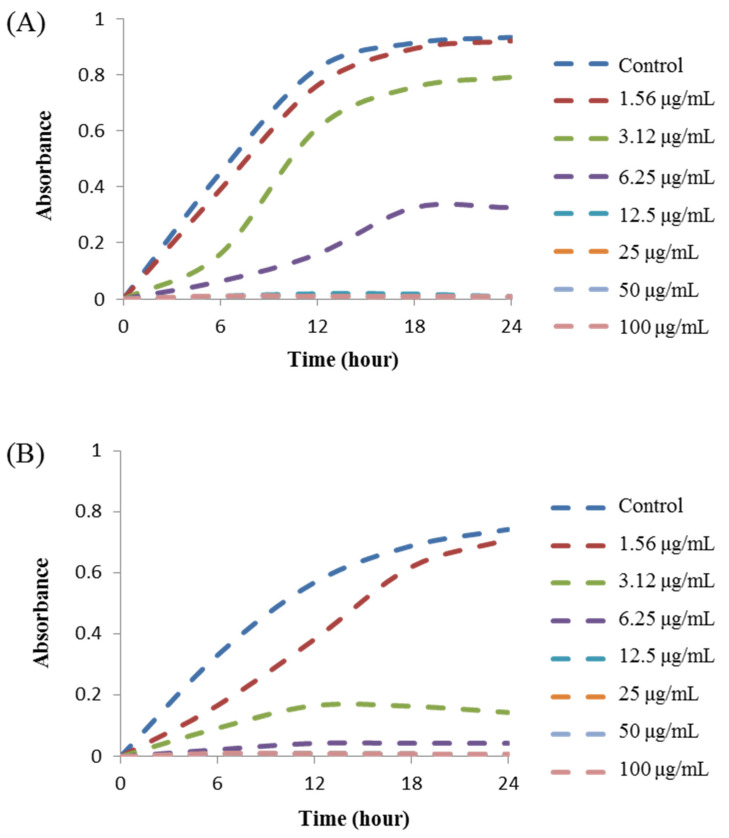
Growth curves of (**A**) *S. typhimurium* and (**B**) *Y. enterocolitica* cultured in MHB with different concentrations of biosynthesized AgNPs to determine MIC.

**Figure 9:**
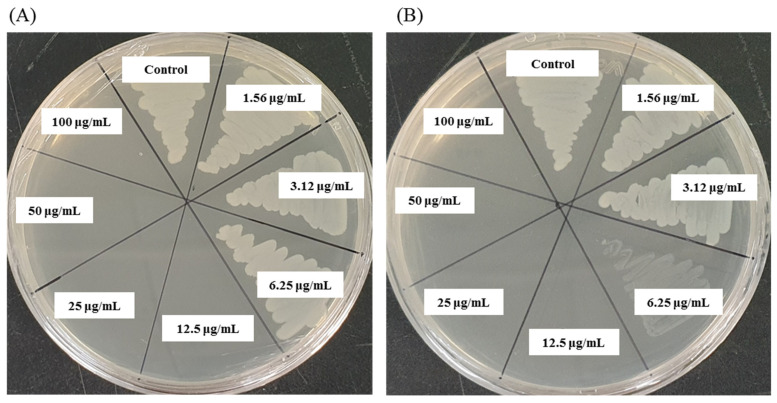
MBC of biosynthesized AgNPs against (**A**) *S. typhimurium* and *(***B**) *Y. enterocolitica*.

The MIC was defined as the lowest concentration of AgNPs that fully inhibited the growth of bacteria. The MIC was determined by a microdilution assay using 96-well plates. The MICs of *Arthrobacter bangladeshi* MAHUQ-56^T^-mediated synthesized AgNPs for *S. typhimurium* and *Y. enterocolitica* were 6.2 and 3.1 ug/mL, respectively (Figure 8, Table 6). These MIC values were significantly lower than those of some other antibacterial agents, including biosynthesized nanoparticles against both *S. typhimurium* and *Y. enterocolitica*. Several studies reported that the MIC values of olive-oil polyphenol extract and Licochalcone A against *S. typhimurium* were 625 and 62.5–1000 ug/mL, respectively [47,48]. Similarly, the MIC values of chitosan nanoparticles and *Parrotia persica* leaf extract against *Y. enterocolitica* were 1500 and 750 ug/mL, respectively [49,50]. MBC is the lowest concentration of antimicrobial agents that fully kills the tested pathogens. The MBC of *Arthrobacter bangladeshi* MAHUQ-56^T^-mediated synthesized AgNPs for both *S. typhimurium* and *Y. enterocolitica* was 12.5 ug/mL (Figure 9, Table 6). This MBC value was also significantly lower than that of some other antibacterial agents against both *S. typhimurium* and *Y. enterocolitica* [47,48,49,50].

### 3.11. Investigation of Morphological Changes by FE-SEM

**Figure 10:**
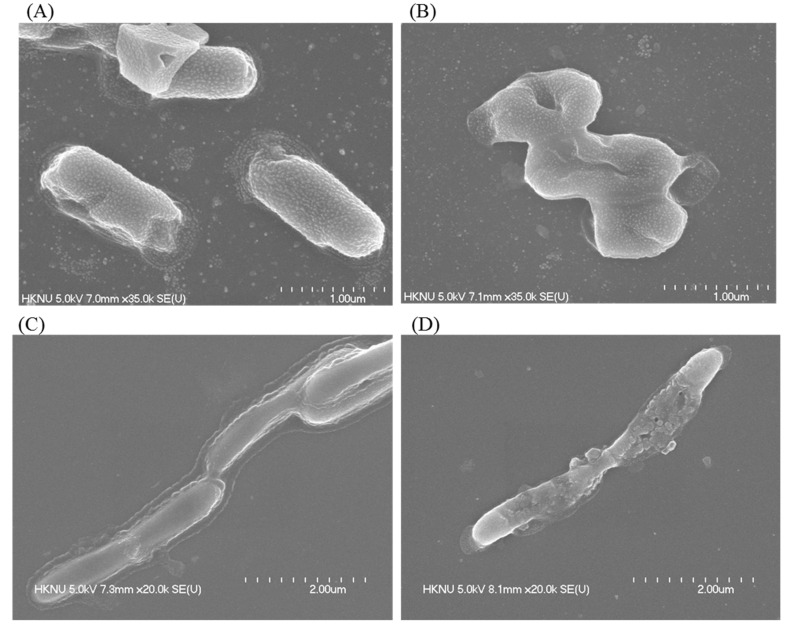
SEM images of (**A**) normal *S. typhimurium* cells, (**B**) 1 × MBC AgNPs treated *S. typhimurium* cells, (**C**) normal *Y. enterocolitica* cells, (**D**) 1 × MBC AgNPs treated *Y. enterocolitica* cells.

Morphological changes in AgNP-treated *S. typhimurium* and *Y. enterocolitica* cells were observed by FE-SEM. FE-SEM images showed the nature and degree of the structural alteration of both tested pathogens (Figure 10). Untreated *S. typhimurium* cells displayed normal morphology and structure without any damage to the cell surface (Figure 10A). However, *S. typhimurium* cells treated with *Arthrobacter bangladeshi* MAHUQ-56^T^-mediated synthesized AgNPs displayed structural changes with abnormal, wrinkled, damaged, and collapsed cell surface and membrane (Figure 10B). Similar results were found for *Y. enterocolitica*. Untreated *Y. enterocolitica* cells exhibited normal rod-shaped structure without any damage to the cell surface (Figure 10C). However, AgNP-treated *Y. enterocolitica* cells exhibited structural changes with abnormal, damaged, and collapsed cell walls and membrane (Figure 10D). The morphological and structural alterations, and damage indicated that the *Arthrobacter bangladeshi* MAHUQ-56^T^-mediated synthesized AgNPs might disturb the normal cellular functions of multidrug-resistant *S. typhimurium* and *Y. enterocolitica,* ultimately leading to the death of cells [44,51].

## 4. Conclusions

The present study described the isolation and characterization of novel bacterial species *Arthrobacter bangladeshi* sp. nov., used for the easy and ecofriendly extracellular synthesis of AgNPs, and investigated its antibacterial efficacy against drug-resistant pathogenic *S. typhimurium* and *Y. enterocolitica*. A facile and convenient extracellular process was used for the biosynthesis of AgNPs using *Arthrobacter bangladeshi* MAHUQ-56^T^. Synthesized AgNPs were characterized by UV-Vis spectroscopy, FE-TEM, XRD, DLS, and FT-IR. Synthesized AgNPs were spherical with a size range of 12–50 nm. FT-IR analysis revealed various biomolecules that may be involved in the synthesis and capping of AgNPs. Biosynthesized AgNPs showed strong antibacterial activity against multidrug-resistant pathogenic *S. typhimurium* and *Y. enterocolitica*. AgNPs showed a ZOI of 18.3 ± 0.6 and 20.4 ± 0.8 mm against *S. typhimurium* and *Y. enterocolitica*, respectively. MIC values of synthesized AgNPs against *S. typhimurium* and *Y. enterocolitica* were 6.2 and 3.1 ug/mL, respectively. The MBC of synthesized AgNPs for both pathogens was 12.5 ug/mL. FE-SEM analysis revealed the morphological and structural alterations, and damage of *S. typhimurium* and *Y. enterocolitica* cells treated by synthesized AgNPs. These changes might disturb the normal cellular functions of multidrug-resistant *S. typhimurium* and *Y. enterocolitica,* ultimately leading to the death of cells. Results of the present study suggest that novel bacterial species *Arthrobacter bangladeshi* MAHUQ-56^T^ could be used for the rapid and mass production of AgNPs, and the synthesized AgNPs could be used as an alternative antibacterial agent to treat multidrug-resistant *S. typhimurium* and *Y. enterocolitica*.

## Figures and Tables

**Table 1:** Differential characteristics of *Arthrobacter bangladeshi* MAHUQ-56^T^ and phylogenetically closely related species.

Characteristics	1	2	3	4	5	6
Isolation source	Soil of rice field	Pokkali rice	Sewage	Filtration substrate from trass	Creosote-contaminated soil	Marine sediments
Cell morphology	Rod	Rod	Rod–coccus	Rod–coccus	Rod–coccus	Rod–coccus
Colony color	Milky white	Yellow	Creamy white	Light yellow	Cream to yellow	Cream to light grey
Growth temperature (°C)	10–40	18–37 ^a^	5–37 ^b^	5–40 ^c^	4–37 ^d^	10–45 ^e^
Growth pH	5.0–10.0	5.5–8.0 ^a^	6.0–10.0 ^b^	6.0–11.0 ^c^	6.5–8.5 ^d^	5.0–9.0 ^e^
**Hydrolysis of:**						
Gelatin (API 20 NE)	-	-	-	+	-	-
Urea (API 20 NE)	-	-	-	W	-	-
**Enzyme activity (API ZYM):**						
Esterase (C4)	+	+	W	+	W	+
Alkaline phosphatase	+	+	-	+	W	+
Esterase lipase (C8)	W	+	-	+	W	+
Acid phosphatase	+	-	+	+	W	-
Valine arylamidase	W	+	-	W	W	+
Trypsin	+	+	-	+	W	-
*β*-glucuronidase	+	+	+	+	+	-
*a*-glucosidase	+	+	+	+	W	-
*β*-galactosidase	+	+	+	+	W	-
*a-fucosidase*	+	+	+	-	-	-
**Assimilation of (API 20 NE):**						
D-glucose	+	-	+	+	-	_+_
D-maltose	+	+	+	-	-	+
D-mannose	+	-	+	-	-	+
D-mannitol	+	+	+	-	W	-
Malic acid	+	+	+	W	W	+
DNA G + C content (mol%)	66.0	64.0 ^a^	64.4 ^b^	70.8 ^c^	65.7 ^d^	61.3 ^e^

Strains: 1, *A. bangladeshi* MAHUQ-56^T^; 2, *A. pokkalii* KCTC 29498^T^; 3, *P. defluvii* KCTC 19209^T^; 4, *P. niigatensis* CCTCC AB 206012^T^; 5, *P. phenanthrenivorans* DSM 18606^T^ and 6, *P. enclensis* DSM 25279^T^. All data were obtained in this study, except ^a–d^ and ^e^, which were taken from Krishnan et al. [4], Kim et al. [5], Ding et al. [6], Kallimanis et al. [7], and Dastager et al. [8], respectively. All strains were aerobic and nonmotile. All strains were positive for catalase, reduction of nitrate, leucine arylamidase and naphthol-AS-BI-phosphohydrolase. All strains were negative for oxidase. +, positive; W, weakly positive; -, negative.

**Table 2:** Genome sequence features of *Arthrobacter bangladeshi* MAHUQ-56^T^.

Features	Strain MAHUQ-56^T^
Accession no.	JAIFZQ000000000
Biosample	SAMN20721218
BioProject	PRJNA754020
Total sequence length (nt)	4,566,112
Scaffold N50	360,503
Scaffold N75	196,969
Number of scaffold	26
Sequencing method	de novo (illumina XTen)
Annotation pipeline	NCBI Prokaryotic Genome
DNA G + C content (mol%)	66.0
Total genes	4233
Genes (coding)	4125
Number of RNAs	60
tRNAs	51
rRNAs	6

**Table 3:** Cellular fatty acid composition of *Arthrobacter bangladeshi* MAHUQ-56^T^ and phylogenetically closely related species.

Fatty Acid	1	2	3	4	5	6
C_14:0_	2.3	1.4	1.5	1.3	1.6	2.0
C_14_._0 iso_	3.5	1.6	3.4	1.5	ND	2.2
C_15:0 iso_	11.9	8.1	13.8	7.8	15.1	14.5
C_15:0 anteiso_	47.8	54.5	55.6	52.0	37.7	48.3
C_16:1_ *w*7*c*	ND	ND	ND	ND	2.6	ND
C_16:0 iso_	15.8	8.5	11.9	12.2	16.0	11.2
C_16:0_	3.8	7.2	4.0	6.0	7.9	6.2
C_17:0 iso_	1.6	1.5	1.1	2.0	4.2	2.0
C_17:0 anteiso_	11.7	13.8	5.9	13.9	13.1	11.3

Strains: 1, *A. bangladeshi* MAHUQ-56^T^; 2, *A. pokkalii* KCTC 29498^T^; 3, *P. defluvii* KCTC 19209^T^; 4, *P. niigatensis* CCTCC AB 206012^T^; 5, *P. phenanthrenivorans* DSM 18606^T^ and 6, *P. enclensis* DSM 25279^T^. All data were collected from this study. Tr, trace (less than 1.0%); ND, not detected.

**Table 4:** Number and percentage of chemical elements present in EDX spectrum of *Arthrobacter bangladeshi* MAHUQ-56^T^-mediated synthesized AgNPs.

Element	Weight %	Atomic %
Cu K	50.66	63.54
Ag L	49.34	36.46
Totals	100.00	100.00

**Table 5:** Antibacterial efficacy of *Arthrobacter bangladeshi* MAHUQ-56^T^-mediated synthesized AgNPs against *S. typhimurium* and *Y. enterocolitica*.

Pathogenic Species	Zone of Inhibition (mm)	
	AgNPs (30 μL)	AgNPs (60 μL)
*Salmonella* Typhimurium (ATCC 14028)	11.5 ± 0.5	18.3 ± 0.6
*Yersinia enterocolitica* (ATCC 9610)	13.5 ± 0.7	20.4 ± 0.8

**Table 6:** Minimum inhibitory concentration (MIC) and minimum bactericidal concentration (MBC) of biosynthesized AgNPs against *S. typhimurium* and *Yersinia enterocolitica*.

Pathogenic Species	MIC (μg/mL)	MBC (μg/mL)
*Salmonella Typhimurium* (ATCC 14028)	6.2	12.5
*Yersinia enterocolitica* (ATCC 9610)	3.1	12.5

## Data Availability

Not applicable.

## Supplementary Materials

The following are available online at https://www.mdpi.com/article/10.3390/pharmaceutics13101691/s1, Figure S1: The maximum-likelihood (ML) tree based on 16S rRNA gene sequence analysis showing phylogenetic relationships of strain MAHUQ-56T and the related type strains. Values less than 50 % were not shown. Scale bar, 0.05 substitutions per nucleotide position, Figure S2: Particles size distribution of biosynthesized AgNPs according to intensity (A), number (B) and volume (C), Table S1: dDDH and ANI values between proposed novel strain MAHUQ-56T and the closest type strains, Table S2: The physicochemical properties of AgNPs synthesized by *Arthrobacter bangladeshi* and some other bacterial strains.
