# Supplementary material for: Characterization and Genome Analysis of Arthrobacter bangladeshi sp. nov., Applied for the Green Synthesis of Silver Nanoparticles and Their Antibacterial Efficacy against Drug-Resistant Human Pathogens
Source: Pharmaceutics. 2021 Oct 15;13(10):1691. doi: 10.3390/pharmaceutics13101691 (PMC8538746; doi:10.3390/pharmaceutics13101691)
Supplement: Supplementary file 1 [file pharmaceutics-13-01691-s001.zip › pharmaceutics-1379978-supplementary.pdf]

pharmaceutics

MDPI

# Supplementary Materials: Characterization and Genome Analysis of *Arthrobacter bangladeshi* sp. nov., Applied for the Green Synthesis of Silver Nanoparticles and Their Antibacterial Efficacy against Drug-Resistant Human Pathogens

**Md. Amdadul Huq and Shahina Akter**

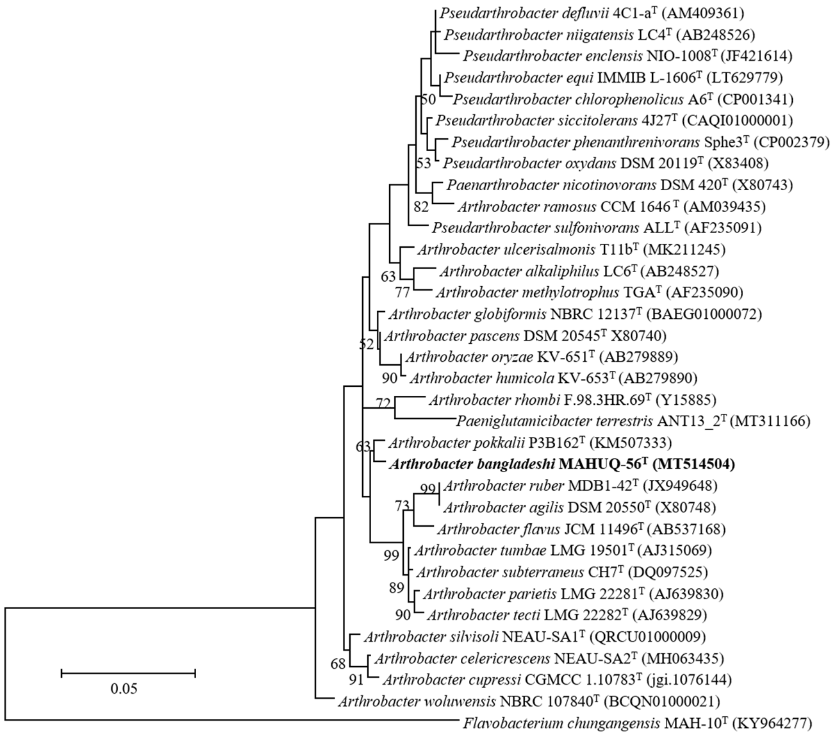


**Figure S1.** The maximum-likelihood (ML) tree based on 16S rRNA gene sequence analysis showing phylogenetic relationships of strain MAHUQ-56 and the related type strains. Values less than 50% were not shown. Scale bar, 0.05 substitutions per nucleotide position.

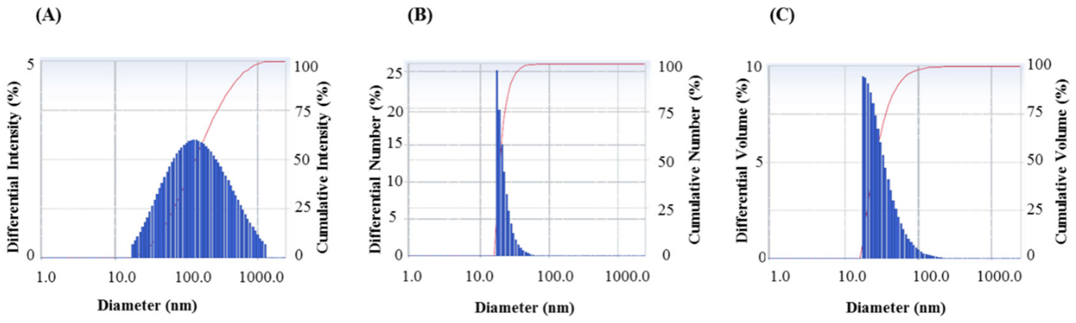


**Figure S2.** Particles size distribution of biosynthesized AgNPs according to intensity (**A**), number (**B**) and volume (**C**).

**Table S1.** dDDH and ANI values between proposed novel strain MAHUQ-56$^T$ and the closest type strains.

| Query genome | Reference genome | Formula 1 | | | | Formula 2 (Recommended) | | | | Formula 3 | | | | ANI (%) |
|---|---|---|---|---|---|---|---|---|---|---|---|---|---|---|
| | | DDH | Model C.I. | Distance | Prob. DDH >= 70% | DDH | Model C.I. | Distanc e | Prob. DDH >= 70% | DDH | Model C.I. | Distanc e | Prob. DDH >= 70% | |
| *Arthrobacter bangladeshi* MAHUQ-56$^T$ (JAIFZQ000000000) | *Arthrobacter pokkalii* GZK-1 (GCA_006715285.1) | 51.9 | [48.4 - 55.3%] | 0.2932 | 16.42 | 27.5 | [25.1–30.0%] | 0.1568 | 0.03 | 44.3 | [41.3 - 47.4%] | 0.4040 | 0.56 | 84.06 Gc=65.86 |
| | *Arthrobacter phenanthrenivorans* Sphe3 (GCA_000189535.1) | 39.3 | [35.9 - 42.8%] | 0.3994 | 1.43 | 24.5 | [22.2 – 27.0%] | 0.1778 | 0.01 | 34.4 | [31.4 - 37.5%] | 0.5062 | 0.02 | 81.53 GC=65.37 |
| | *Arthrobacter enclensis* NIO-1008 (GCA_001457025.1) | 40.8 | [37.5 - 44.3%] | 0.3843 | 2.05 | 24.8 | [22.5 - 27.3%] | 0.1758 | 0.01 | 35.5 | [32.6 - 38.6%] | 0.4926 | 0.03 | 82.02 GC=67.09 |

**Table S2.** dDDH and ANI values between proposed novel strain MAHUQ-56$^T$ and the closest type strains.

| Bacteria used for synthesis of AgNPs | Size of AgNPs | Average hydrodynamic diameter | Polydispersity index | Absorbance peaks found by FTIR analysis | Reference |
|---|---|---|---|---|---|
| *Arthrobacter bangladeshi* | 12-50 nm | 122.5 nm | 0.286 | 3438.60, 2921.32, 2848.50, 2352.20, 2330.50 and 1645.20 $cm^{-1}$ | In the current study |
| *Cedecea* sp. | 10–40 nm | 115.9 nm | 0.39 | 3241.87, 2915.24, 2848.56, 2113.44, 1999.70, 1922.51, 1621.15, 1514.19, 1444.29 and 1007.59 $cm^{-1}$ | [43] |
| *Paenarthrobacter nicotinovorans* | 13–27 nm | 44.2 nm | 0.443 | 3420.90, 2921.69, 2851.34, 2360.21, 2341.64, 1653.02, and 1033.62 $cm^{-1}$ | [45] |
| *Terrabacter humi* | 6-24 nm | Not available | Not available | 3444.65, 2917.98, 2847.83, 2360.10, 2342.18, 1635.91, 1374.25 and 1175.36 $cm^{-1}$ | [46] |
